# Involvement of Inflammatory Cytokines in Antiarrhythmic Effects of Clofibrate in Ouabain-Induced Arrhythmia in Isolated Rat Atria

**DOI:** 10.1155/2016/9128018

**Published:** 2016-02-10

**Authors:** Somayeh Moradi, Vahid Nikoui, Muhammad Imran Khan, Shayan Amiri, Farahnaz Jazaeri, Azam Bakhtiarian

**Affiliations:** ^1^Department of Pharmacology, School of Medicine, Tehran University of Medical Sciences, Tehran, Iran; ^2^Razi Institute for Drug Research, Iran University of Medical Sciences, Tehran, Iran; ^3^Department of Pharmacology, Faculty of Medicine, Iran University of Medical Sciences, Tehran, Iran; ^4^Department of Pharmacology, School of Medicine, International Campus, Tehran University of Medical Sciences, Tehran, Iran; ^5^Experimental Medicine Research Center, Tehran University of Medical Sciences, Tehran, Iran

## Abstract

Considering the cardioprotective and anti-inflammatory properties of clofibrate, the aim of the present experiment was to investigate the involvement of local and systemic inflammatory cytokines in possible antiarrhythmic effects of clofibrate in ouabain-induced arrhythmia in rats. Rats were orally treated with clofibrate (300 mg/kg), and ouabain (0.56 mg/kg) was administered to animals intraperitoneally. After induction of anesthesia, the atria were isolated and the onset of arrhythmia and asystole was recorded. The levels of inflammatory cytokines in atria were also measured. Clofibrate significantly postponed the onset of arrhythmia and asystole when compared to control group (*P* ≤ 0.05 and *P* ≤ 0.01, resp.). While ouabain significantly increased the atrial beating rate in control group (*P* ≤ 0.05), same treatment did not show similar effect in clofibrate-treated group (*P* > 0.05). Injection of ouabain significantly increased the atrial and systemic levels of all studied inflammatory cytokines (*P* ≤ 0.05). Pretreatment with clofibrate could attenuate the ouabain-induced elevation of IL-6 and TNF-*α* in atria (*P* ≤ 0.01 and *P* ≤ 0.05, resp.), as well as ouabain-induced increase in IL-6 in plasma (*P* ≤ 0.05). Based on our findings, clofibrate may possess antiarrhythmic properties through mitigating the local and systemic inflammatory factors including IL-6 and TNF-*α*.

## 1. Background

The fibrate class of hypolipidemic drugs is used extensively in treatment of metabolic syndrome in which hyperlipidemia and hypertension are most prominent manifestations of this disorder. Fibrates are ligands of the peroxisome proliferator-activated receptors (PPARs) [1, 2]. These receptors are ligand-dependent transcription factors and belong to the nuclear steroid/thyroid/retinoic acid receptor superfamily [3, 4]. PPARs consisted of three isotypes including *α*, *β*, and *γ*. PPAR-*α* possesses an important role in lipid metabolism [5]. PPAR-*α* is predominantly expressed in tissues with high fatty acid oxidation rate including heart, liver, and kidney [6]. It has been shown that fibrates protect heart against experimental ischemia/reperfusion injury in animals through PPARs [7, 8]. Interestingly, previous studies have shown that cardioprotective effects of fibrates are not observed in PPAR-*α* knockout mice, indicating that PPAR-*α* plays a critical role in cardioprotective effects of fibrates [7, 8]. Since majority of hyperlipidemic patients are suffering from comorbid cardiovascular diseases, it is clear that many of the cardiovascular patients use clofibrate. It has been demonstrated that PPAR-*α* agonists have anti-inflammatory properties [9–12], and inflammatory cytokines have been reported to be involved in atrial and ventricular arrhythmias [13–15]. Therefore, the aim of present study was to investigate the effects of clofibrate on ouabain-induced arrhythmia in isolated rat atria and involvement of local and systemic inflammatory cytokines.

## 2. Material and Methods

### 2.1. Animals

Male Wistar albino rats (body weight 250–280 g) were obtained from the Department of Pharmacology and Comparative Biology Unit (Tehran University of Medical Sciences). Animals were housed under the standard laboratory conditions, temperature 22 ± 2°C, humidity 70%–80%, and 12 h light-dark cycle, and have* ad libitum* access to food and water. All experiments were conducted in Tehran University of Medical Sciences in accordance with the recommendations of the ethics committee on animal experimentation of the medical school.

### 2.2. Chemicals

All materials were purchased from Merck (Germany), unless noted otherwise. Clofibrate was a gift from Zahravi Pharmaceutical Company, Iran. Ouabain and enzyme-linked immunosorbent assay (ELISA) kit for measurement of IL-6 and TNF-*α* were provided from Sigma-Aldrich (St. Louis, MO, USA). ELISA kit for measurement of IL-1*β* was purchased from Abcam, UK.

### 2.3. Experimental Plan

In order to study the isolated atria, 12 rats were randomly divided into 2 equal groups. First group (treatment) received clofibrate (300 mg/kg dissolved in olive oil 1 mL/kg, orally) once daily for 14 days, while the second group (control) received only olive oil (1 mL/kg, orally) once daily for 14 days. For measuring the inflammatory cytokines, 12 rats were divided into three equal groups. First 2 groups received only olive oil (1 mL/kg, orally) once daily for 14 days, while third group was given clofibrate (300 mg/kg, dissolved in olive oil 1 mL/kg, orally) once daily for 14 days. From the days 12th to 14th, first group received normal saline, 1 mL/kg, intraperitoneally (i.p.) once daily, whereas the second and third groups were injected by ouabain dissolved in normal saline at the dose of 0.56 mg/kg [16] once daily (i.p.) for three consecutive days.

### 2.4. Preparation of Isolated Atria

After induction of anesthesia with ketamine (80 mg/kg, Alfasan, Netherlands) and diazepam (2 mg/kg, Caspian Tamin, Iran) (i.p.), heart was rapidly removed and atria were carefully dissected from the ventricles, attached to a tissue holder, and then were immersed in a tissue bath containing 25 mL of carbogenated (95% O_2_ and 5% CO_2_) modified Krebs solution at 37°C and pH 7.4. The composition of the solution was as follows (mM): NaCl 118.0, KCl 4.7, CaCl_2_ 2.6, MgCl_2_ 1.2, NaH_2_PO_4_ 1.0, NaHCO_3_ 25.0, glucose 11.1, EDTA 0.004, and ascorbic acid 0.11. A preload tension of 1000 mg was applied to the atria and tissues were allowed to equilibrate for 30 min [17]. The rate and force of spontaneous contractions were recorded by the isometric force transducer of PowerLab system (ADInstrument, Australia). Atrial beats and contractile forces were calculated using the LabChart software.

### 2.5. Measurement of IL-1*β*, IL-6, and TNF-*α* in Atria and Plasma

The levels of inflammatory cytokines in atria and plasma were measured by specific ELISA kits. Briefly, each atrium was homogenized in 1 mL of phosphate buffered saline (PBS, pH 7.4) and then centrifuged at 10000 g for 15 min at 4°C. For measurement of plasma levels of cytokines, blood samples were taken and centrifuged at 4000 g for 15 min at 4°C. IL-1*β*, IL-6, and TNF-*α* levels were measured by adding the sample supernatant and kit reagents to plate wells. Absorbance was read at 450 nm using a plate reader (Synergy HT, Biotek, USA), and the contents of IL-1*β*, IL-6, and TNF-*α* in atria and plasma were calculated and reported as ng/mL.

### 2.6. Statistical Analysis

Statistical analyses were carried out using GraphPad Prism 5 software (San Diego, CA, USA). The results are presented as mean ± SEM. Unpaired Student's *t*-test was carried out to compare the time of onset of arrhythmia or asystole as well as atrial beating rate and contractile force between treatment and control groups. Paired Student's *t*-test was used to detect the effects of ouabain on atrial beating rate and contractile force within groups. Comparison of inflammatory cytokines between three groups was done by one-way analysis of variance (ANOVA) followed by Tukey's* post hoc* test. *P* values less than 0.05 were considered statistically significant.

## 3. Results

### 3.1. Study of Isolated Atria

**Figure 1:**
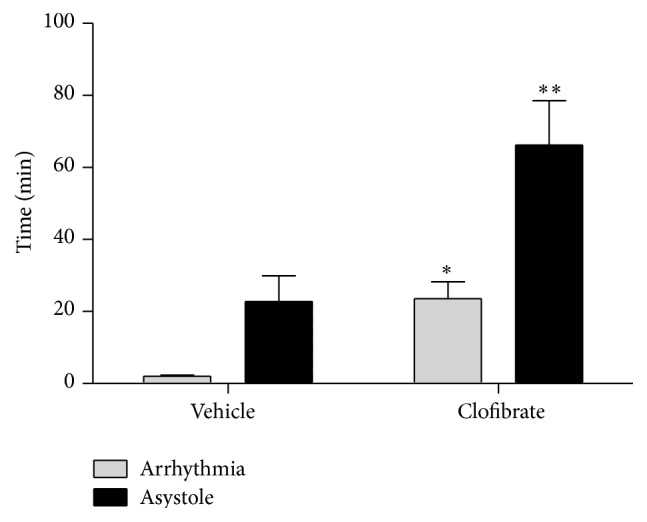
Time of onset of arrhythmia and asystole after ouabain incubation in vehicle- and clofibrate-treated groups. Data are shown as mean ± SEM. Six rats were used in each group. ^*∗*^ *P* ≤ 0.05 compared to arrhythmia of vehicle group; ^*∗∗*^ *P* ≤ 0.05 compared to asystole of vehicle group.

Clofibrate significantly postponed time of onset of arrhythmia (23.57 ± 4.69 min) rather than control group (2.04 ± 0.27 min, *P* ≤ 0.05). Also, we detected a significant increase in the onset time of asystole in the treatment group (66.19 ± 12.33 min), while this time for control group was 22.77 ± 7.17 min (*P* ≤ 0.01, Figure 1).

**Figure 2:**
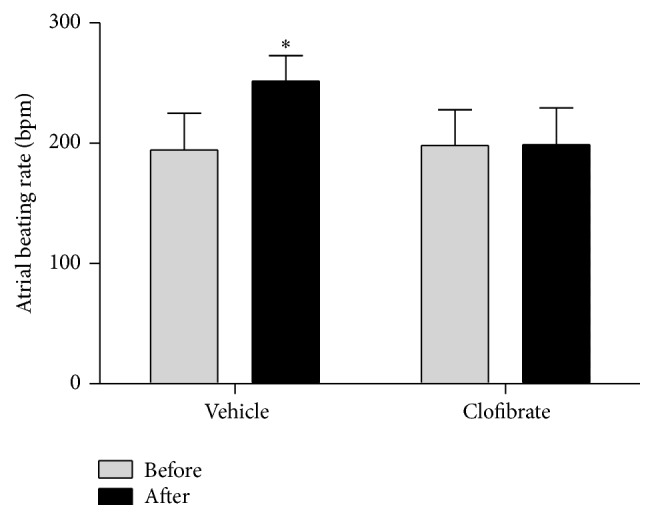
Atrial beating rates before and after incubation of ouabain in vehicle- and clofibrate-treated groups. Data are shown as mean ± SEM. Six rats were used in each group. ^*∗*^ *P* ≤ 0.05 compared to before time of vehicle group.

Treating with ouabain significantly increased the atrial beating rate in the control group (194 before and 251 beats per min after ouabain incubation, *P* ≤ 0.05), while such effect was not observed in treatment group (198 before and 199 beats per min after ouabain incubation, *P* > 0.05, Figure 2).

**Figure 3:**
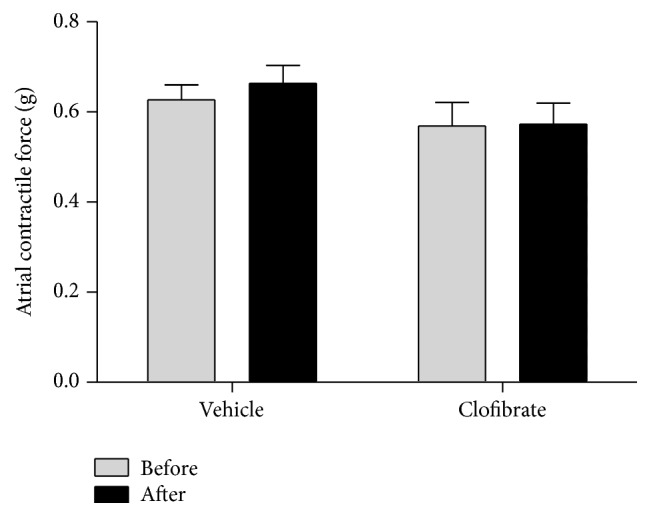
Contractile force before and after incubation of ouabain in vehicle- and clofibrate-treated groups. Data are shown as mean ± SEM. Six rats were used in each group.

**Figure 4:**
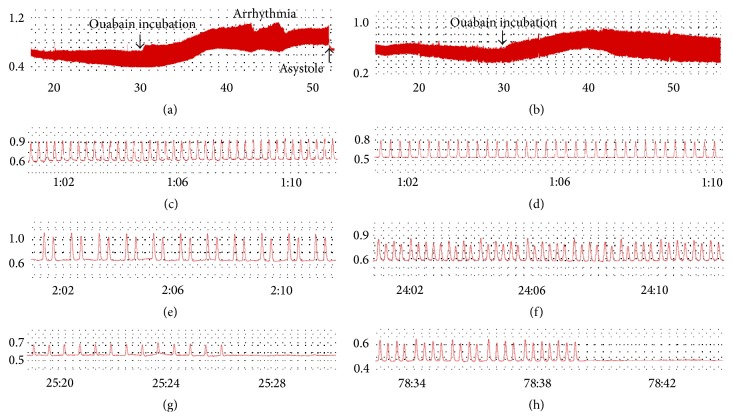
A general comparison in chronotropic and inotropic features between vehicle- and clofibrate-treated groups. Complete records of isolated atrial beats of vehicle-treated (a) and clofibrate-treated (b) groups. In vehicle-treated group (a), ouabain-induced arrhythmia is obvious and asystole happens after arrhythmia. The severity of arrhythmia in clofibrate-treated group is lesser and, in some samples (b), no asystole was seen until several hours. Atrial beatings before incubation of ouabain in vehicle-treated (c) and clofibrate-treated (d) groups. As shown, atrial beatings and contractile force are similar in both groups. Ouabain-induced arrhythmia in vehicle-treated (e) and clofibrate-treated (f) groups. The shape of arrhythmia in vehicle-treated group is bigeminy (twin spikes with strong force), which is the typical manifestation of ouabain-induced arrhythmia (a). In clofibrate-treated group (b), spikes of arrhythmia are weak and irregular, and it happens much later than vehicle-treated group. Ouabain-induced asystole in vehicle-treated (g) and clofibrate-treated (h) groups. Time of onset of asystole in clofibrate-treated group (h) is later than vehicle-treated group.

Treatment with ouabain had no effect on contractile force in both groups (0.627 g before and 0.663 g after ouabain incubation for the control group, *P* > 0.05, and 0.569 g before and 0.573 g after ouabain incubation for the treatment group, *P* > 0.05, Figure 3). There was also no significant difference in atrial beating rate and contractile force between the two groups before incubation with ouabain (*P* > 0.05 for atrial beating rate, Figure 2, and *P* > 0.05 for contractile force, Figure 3).  Figure 4 represents a general comparison in chronotropic and inotropic features between both groups, but the intensity of arrhythmia in the control group (a) was greater than the treatment group (b). Time of onset of asystole was also observed earlier in control group (a). The shapes of the spikes were normal in both groups before incubation of ouabain (c, d).  Figure 4(e) obviously shows the ouabain-induced arrhythmia in control group, while the severity of this arrhythmia is smaller in treatment group (f).

### 3.2. Levels of IL-1*β*, IL-6, and TNF-*α* in Atria and Plasma

**Figure 5:**
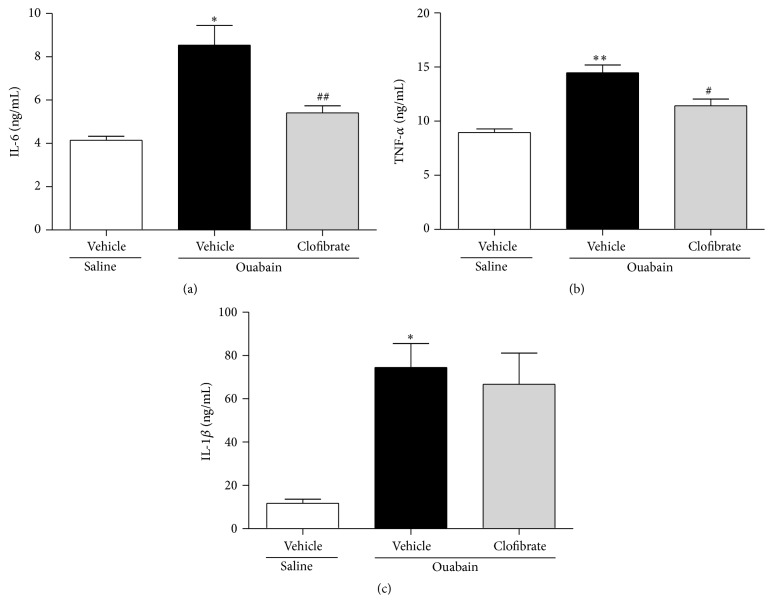
Atrial levels of IL-6 (a), TNF-*α* (b), and IL-1*β* (c) in saline- and ouabain-injected vehicle- and clofibrate-treated groups. Data are shown as mean ± SEM. Four rats were used in each group. ^*∗*^ *P* ≤ 0.01 and ^*∗∗*^ *P* ≤ 0.001 compared to saline-injected vehicle-treated group; ^#^ *P* ≤ 0.05 and ^##^ *P* ≤ 0.01 compared to ouabain-injected vehicle-treated group.

As shown in Figure 5, injection of ouabain significantly increased the atrial levels of all studied inflammatory cytokines (*P* ≤ 0.01 for IL-1*β* and IL-6 and *P* ≤ 0.001 for TNF-*α*). Pretreatment with clofibrate attenuated the ouabain-induced elevation of IL-6 and TNF-*α* in atria (*P* ≤ 0.01 and *P* ≤ 0.05, resp., Figures 5(a) and 5(b)), while it failed to decrease the ouabain-induced elevation in IL-1*β* (*P* > 0.05, Figure 5(c)).

**Figure 6:**
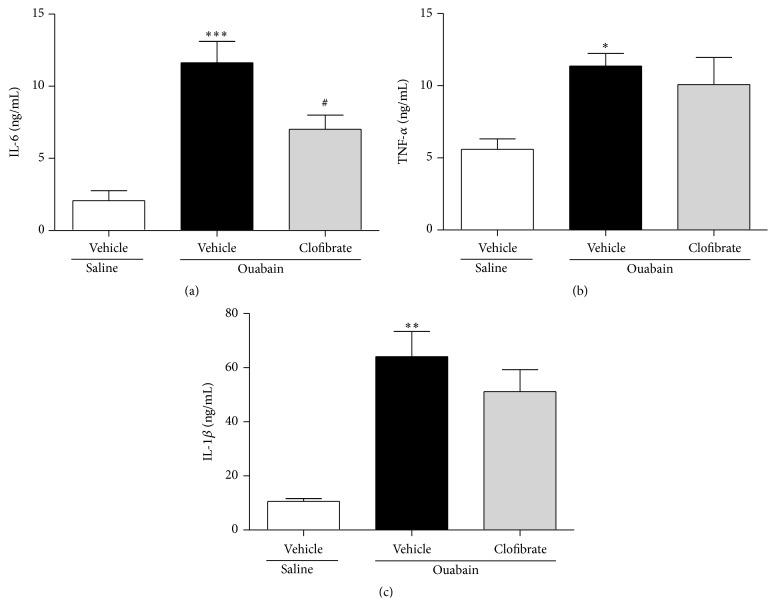
Plasma levels of IL-6 (a), TNF-*α* (b), and IL-1*β* (c) in saline- and ouabain-injected vehicle- and clofibrate-treated groups. Data are shown as mean ± SEM. Four rats were used in each group. ^*∗*^ *P* ≤ 0.05, ^*∗∗*^ *P* ≤ 0.01, and ^*∗∗∗*^ *P* ≤ 0.001 compared to saline-injected vehicle-treated group; ^#^ *P* ≤ 0.05 compared to ouabain-injected vehicle-treated group.

Similarly, ouabain boosted the plasma levels of all inflammatory cytokines significantly (*P* ≤ 0.001, *P* ≤ 0.05, and *P* ≤ 0.01 for IL-1*β*, IL-6, and TNF-*α*, resp., Figure 6). Nevertheless, pretreatment with clofibrate could only reverse the ouabain-induced elevation of IL-6 in plasma (*P* ≤ 0.05, Figure 6(a)), whereas it did not reduce the ouabain-induced increase in TNF-*α* and IL-1*β* levels in plasma (*P* > 0.05, Figures 6(b) and 6(c)).

## 4. Discussion

Cardiac arrhythmias are life-threatening medical conditions. As most antiarrhythmic drugs have serious adverse effects, finding safer antiarrhythmic agents with less side effects has been suggested in the literature. In the present study, we investigated the effects of clofibrate on ouabain-induced arrhythmia in isolated rat atria. For this purpose, induction of arrhythmia and measurement of inflammatory cytokines were performed in rats given ouabain or vehicle. Our results showed that clofibrate, as a PPAR-*α* agonist, succeeded to induce antiarrhythmic effects by delaying the onset and reducing the intensity of ouabain-induced arrhythmias and reduction of ouabain-induced elevation of inflammatory cytokines especially IL-6 in isolated rat atria. In addition, clofibrate alleviated the positive chronotropic effect of ouabain.

It has been reported that ouabain induces the production of proinflammatory cytokines. For example, Matsumori et al. showed that incubation of ouabain increased the levels of IL-1*α*, IL-1*β*, and IL-6 in cultured human peripheral blood mononuclear cells [18]. They also reported that ouabain is able to increase the production of TNF-*α* [19]. In line with previous studies, we showed that ouabain increased proinflammatory cytokines (IL-1*β*, IL-6, and TNF-*α* in both of atria and plasma, Figures 5 and 6), in the atria of treated rats. Furthermore, we demonstrated that clofibrate decreased the inflammatory cytokines in atria (IL-6 and TNF-*α*, Figures 5(a) and 5(b)) and plasma (IL-6, Figure 6(a)). In this regard, Jiang et al. reported that PPAR-*γ* agonists could suppress phorbol ester-induced elevation of IL-1*β*, IL-6, and TNF-*α* [20]. Additionally, it has been shown that PPAR-*γ* agonists improve insulin resistance through inhibiting the effect of TNF-*α* in adipocytes [21]. Although these reports mentioned the protective effects of PPAR-*γ* receptor agonists against inflammatory cytokines, we found such effects in case of PPAR-*α* receptor agonist.

Inflammatory cytokines play a key role in mediating the inflammatory responses under pathologic conditions. TNF-*α* and IL-1 stimulate the release of other inflammatory cytokines such as IL-6 [22]. Evidence indicates that atrial fibrillation after coronary artery bypass grafting is associated with increase in IL-6 in patients [23, 24]. Similar studies have reported that atrial fibrillation results in sequestration of inflammatory cytokines in the heart [13, 14]. With IL-6, there are numerous published studies that suggest that TNF-*α* (and to some extent IL-1*β*) possess arrhythmogenic properties. In this context, TNF-*α* and IL-1*β* have been reported to boost susceptibility to arrhythmia in rat ventricular myocytes through increase of calcium leakage from the sarcoplasmic reticulum [15]. Moreover, it is frequently reported that overexpression of TNF-*α* in transgenic animals causes various atrial and ventricular arrhythmias [25–28]. Recent evidence suggests that inflammatory factors, mainly TNF-*α*, contribute to pathophysiology of ventricular arrhythmias [29]. These findings justify the results of the present study and strengthened our hypothesis that local and systemic inhibition of inflammatory cytokines modulate antiarrhythmic properties of PPAR-*α* agonist, clofibrate. It seems that the role of IL-6 in mediating the antiarrhythmic properties of clofibrate is more prominent than TNF-*α*, because clofibrate reduced IL-6, locally and systemically, while it only decreased the local elevation of TNF-*α*. On the other hand, the local antiarrhythmic effects of clofibrate appear superior to systemic antiarrhythmic properties.

Several lines of research have indicated that there is a high rate of atrial fibrillation and other kinds of cardiac arrhythmias in metabolic syndrome and lipid metabolism disorders [30, 31]. Evidence shows that pretreatment with PPAR-*α* agonist, clofibrate, can reduce the experimental myocardial infarct size up to 43% in rats [32], and it is well established that cardiac infarction and ischemia can cause the most types of arrhythmias. Tabernero et al. and Yue et al. have also reported that PPAR-*α* agonists protect the heart against the ischemia-reperfusion injury [7, 8]. Since clofibrate modulates lipid metabolism, this effect might also partly explain its antiarrhythmic properties, which could be investigated in future studies. Although we did not use ischemia-reperfusion model for induction of arrhythmia in the present study, clofibrate could significantly postpone the time of onset of arrhythmia and asystole in ouabain-induced arrhythmias.

## 5. Conclusions

It is concluded that clofibrate might possess antiarrhythmic properties in reducing the cardiac arrhythmias. It seems that clofibrate shows this beneficial effect through the modulation of some local and systemic inflammatory cytokines including IL-6 and TNF-*α*.
